# 100 Burn Causes Aberrant Macrophage Polarity Conversion in Skeletal Muscles, Which Is Mitigated by Auto/mitophagy-targeted Therapy

**DOI:** 10.1093/jbcr/irac012.103

**Published:** 2022-03-23

**Authors:** Yoh Sugawara, Mohammed A Khan, Hiroyuki Morinaga, Jeevendra Martyn, Shingo Yasuhara

**Affiliations:** Shriners Hospital for Children, Boston, Boston, Massachusetts; Shriners Hospitals for Children, Boston, Massachusetts; Kyorin University, Tokyo, Tokyo; Shriners Hospitals for Dallas, Boston, Massachusetts; Shriners Hospital for Dallas, Boston, Massachusetts

## Abstract

**Introduction:**

Major burn injury (BI) is associated with excessive inflammation. We have reported autophagy/mitophagy response defect in skeletal muscles in BI, leading to diminished homeostasis capability of cells and mitochondria. Macrophages play central roles in the process of wound healing, from causing inflammation, clearing cell debris, to coordinating tissue repair. Classically, M1 macrophages are considered as pro-inflammatory phenotype, and M2 as anti-inflammatory/pro-regeneration phenotype, though complexity of constituent of macrophage subpopulations, being increasingly recognized. The relationship between BI-induced autophagy/mitophagy defect and the regulation of macrophage polarity in BI has not been studied so far.

**Methods:**

Body trunk BI covering 30% of body surface area or sham-bun (SB) treatment was administered to wild type mice. At post burn day (PBD) 5, mechanical injury was created in sternomastoid muscles (STM) by pressure injection of saline into the tissue ( >3x fracture toughness) to study effect of BI on the wound healing process in muscles. STMs were harvested at post surgical day (PSD)1, 3, 7 and 14, and cryosectioned for immunohistochemistry against satellite cells (pax7), and macrophages (CD86 for M1, CD206 for M2, and F4/80 for general). As a therapeutic approach, we tested the effect of auto/mitophagy modulating drug, trehalose, whose pro-auto/mitophagy effect has been attributed to mitigation of the blocked flux of auto/mitophagy (due to BI) in our separate studies.

**Results:**

SB control group showed both total and M1 macrophage numbers chronologically decreasing from PDS1 towards PSD14. BI caused delay in the subsiding of the macrophage infiltration. The greater majority of the macrophage in BI was CD86 (+) M1 phenotype, confirming the pro-inflammatory traits in BI (+46.4% of SB at PSD7). Contrarily, CD206 (+) M2 macrophage started from low numbers at PSD1 and increased towards PSD7 and 14 in SB. In BI, however, M1/M2 conversion was markedly delayed (-59% of SB at PSD7), which was mitigated by trehalose (+134% of BI at PSD7). Pax7 (+) muscle satellite cells increased from PSD1 towards PSD14 in SB, but the response was prominently defective in BI, and was normalized by trehalose.

**Conclusions:**

M1 to M2 conversion observed in SB was defective in BI with concomitant defect in satellite cell proliferation. Autophagy/mitophagy treatment drug, trehalose mitigated the phenotype conversion defect in macrophage.

